# Supplementary material for: Suppression of Rice Osa-miR444.2 Improves the Resistance to Sheath Blight in Rice Mediating through the Phytohormone Pathway
Source: Int J Mol Sci. 2023 Feb 11;24(4):3653. doi: 10.3390/ijms24043653 (PMC9963240; doi:10.3390/ijms24043653)
Supplement: Supplementary file 1 [file ijms-24-03653-s001.zip › ijms-2189027-supplementary.pdf]

International Journal of
Molecular Sciences

MDPI

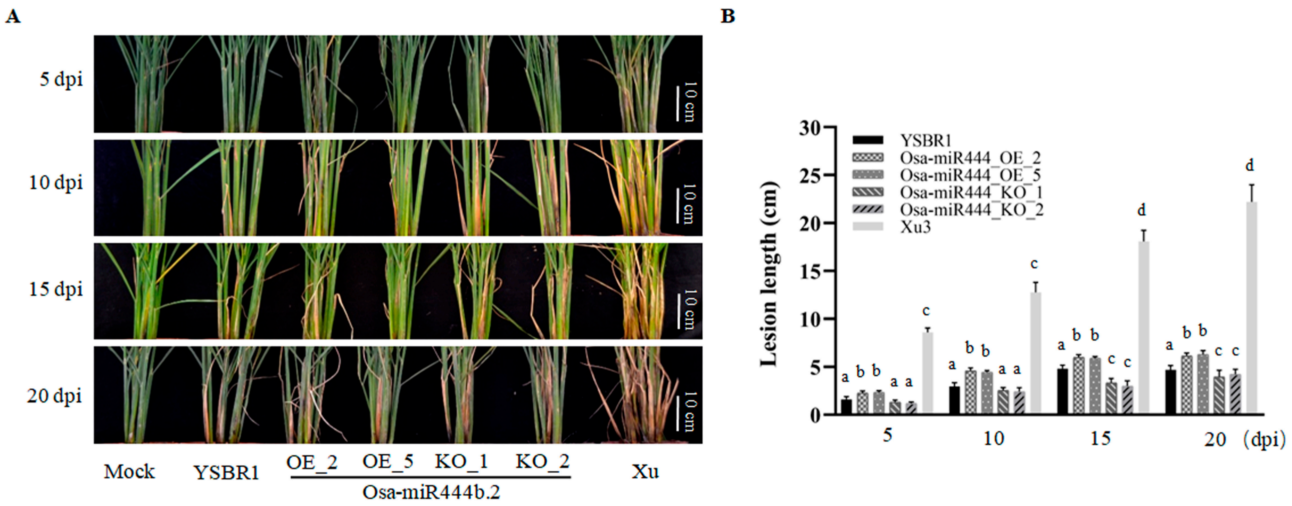

Figure S1 Osa-miR444b.2 enhanced rice susceptibility to sheath blight in YSBR1.

A Disease phenotypes at different time points after infected by *R. solani*. Scale bar, 10 cm. **B** The lesion length of knockout materials at different time points after infected by *R. solani*. The lesion length of overexpression materials at different time points after infected by *R. solani*. a presents the largest average, bcd present significance (P<0.01).

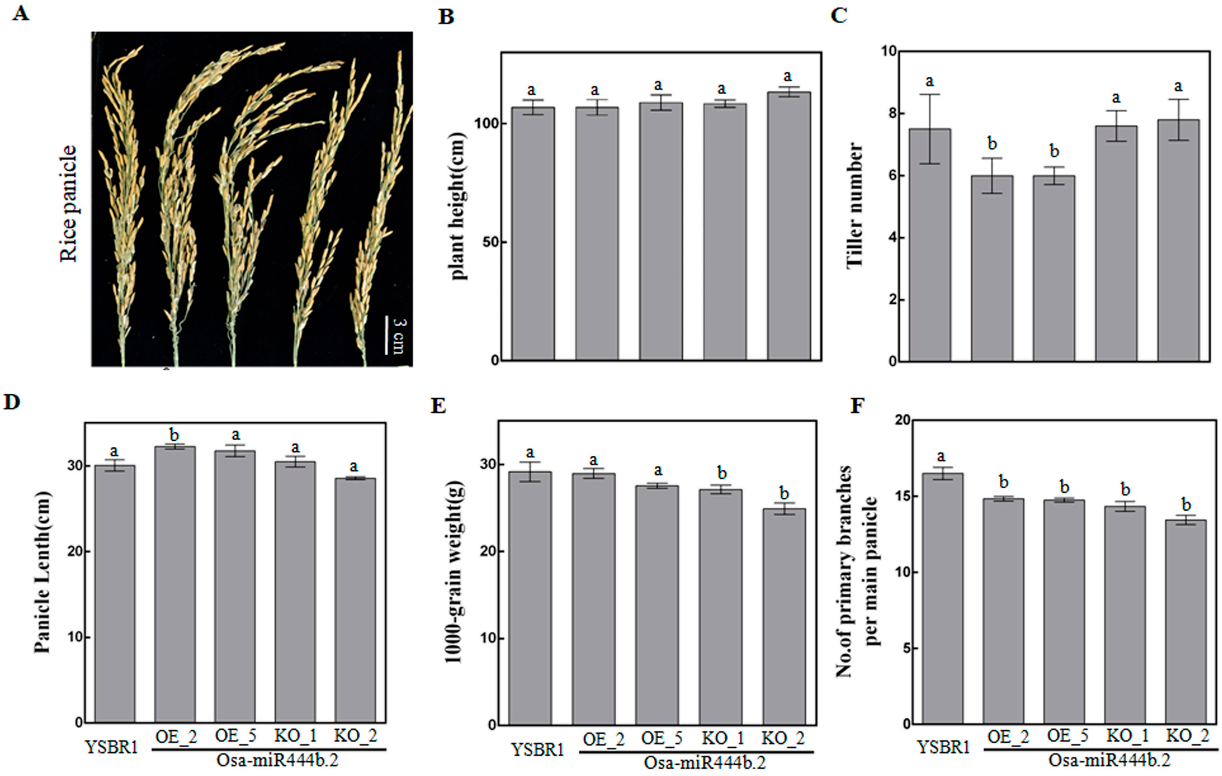

Figure S2 Osa-miR444b.2 expression level affects the agronomic traits in YSBR1.

A Panicle morphology of Osa-miR444b.2 knockout and overexpression materials. **B** Statistics of the plant height. **C** Statistics of the tiller number. **D** Statistics of the panicle length. **E** Statistics of the 1000-grains weight. **F** Statistics of the number of primary branches per main panicle. a presents the largest average, b present significance (P<0.01).

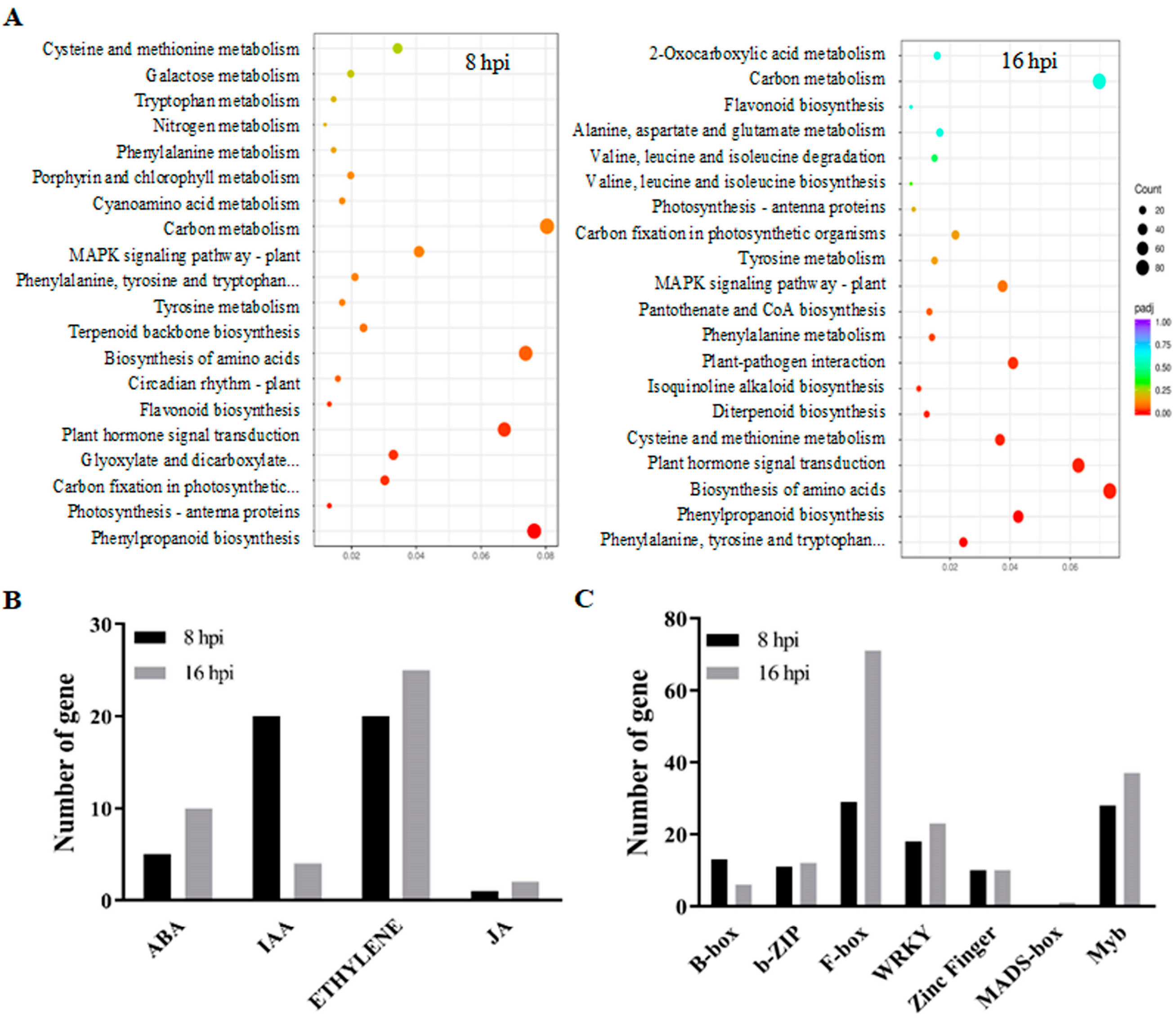

Figure S3 Osa-miR444b.2 participates in the response to *R. solani*infection by regulating plant hormone signal transduction pathway and transcription factors.

A Top 20 enriched KEGG pathways of differentially expressed genes in differential time. **B** The number of differentially expressed genes in plant hormone signal transduction. **C** The number of differentially expressed genes of TFs.

Table S1 Table Primers used in this study.

Purpose	ID	Sequence（5′→3′）
Reverse	miR444- stemloop	GTCGTATCCAGTGCAGGGTCCGAGGTA
Transcription	primer	TTCGCACTGGATACGACAAGCTT
qRT-PCR	miR444-RT-F	GCGGCGGTGCAGTTGTTGTCTC
	Universal reverse	GTGCAGGGTCCGAGGT
	primer	
	*Os*18s RNA-F	CTACGTCCCTGCCCTTTGTACA

*Os*18s RNA-R	ACACTTCACCGGACCATTCAA
Os12g26290 RT-F	GACCTCCGTGATGTTTTCTC
Os12g26290 RT-R	CAGCATTATGGGGGTCGTTG
Os07g03730 RT-F	CGGAGAAGCAGTGGTACGAC
Os07g03730 RT-R	GCGAGTAGTTGCAGGTGATG
Os08g38990 RT-F	GTGCCAACCAGAAATCAAGC
Os08g38990 RT-R	CAGTGGTAGGAGAAGGTTGT
Os12g36850 RT-F	GCTGTGGAAGGTCTGCTTGG
Os12g36850 RT-R	CCAACACCTCAACCTTTAGC
Os11g37950 RT-F	CTACGTGGGACGCTAACAAG
Os11g37950 RT-R	CTTGGTGAAGACGGTGTCCC
Os09g25070 RT-F	CGTGACAACCCCTACCCTAG
Os09g25070 RT-R	CCGACGAGTTGATGGAGATG
Os02g33820 RT-F	GCGTGGTGGACTACGACAAG
Os02g33820 RT-R	CGTGCTTCTTGGCGTCCTTC
Os01g72910 RT-F	GCAAGGAGGAGAAGCACCAC
Os01g72910 RT-R	GTGCTTCTTGGCTTCCTTCT
Os01g32380 RT-F	GACGTGGACAGGTTCTACGC
Os01g32380 RT-R	GCTGTCCACCTTCTTCTCCG
Os03g18850 RT-F	TCAACTTCACCTCAGCCATG
Os03g18850 RT-R	GTGATCTCGTCCTTCACCTC
